# Tocopherols and Tocotrienols—Bioactive Dietary Compounds; What Is Certain, What Is Doubt?

**DOI:** 10.3390/ijms22126222

**Published:** 2021-06-09

**Authors:** Kacper Szewczyk, Aleksandra Chojnacka, Magdalena Górnicka

**Affiliations:** Institute of Human Nutrition Sciences, Warsaw University of Life Sciences (SGGW-WULS), 159C Nowoursynowska Street, 02-787 Warsaw, Poland; kacper_szewczyk@sggw.edu.pl (K.S.); aleksandrachojnacka95@o2.pl (A.C.)

**Keywords:** tocopherols, tocotrienols, vitamin E metabolites, α-tocopherol, bioaccessibility, bioavailability, bioactivity

## Abstract

Tocopherols and tocotrienols are natural compounds of plant origin, available in the nature. They are supplied in various amounts in a diet, mainly from vegetable oils, some oilseeds, and nuts. The main forms in the diet are α- and γ-tocopherol, due to the highest content in food products. Nevertheless, α-tocopherol is the main form of vitamin E with the highest tissue concentration. The α- forms of both tocopherols and tocotrienols are considered as the most metabolically active. Currently, research results indicate also a greater antioxidant potential of tocotrienols than tocopherols. Moreover, the biological role of vitamin E metabolites have received increasing interest. The aim of this review is to update the knowledge of tocopherol and tocotrienol bioactivity, with a particular focus on their bioavailability, distribution, and metabolism determinants in humans. Almost one hundred years after the start of research on α-tocopherol, its biological properties are still under investigation. For several decades, researchers’ interest in the biological importance of other forms of vitamin E has also been growing. Some of the functions, for instance the antioxidant functions of α- and γ-tocopherols, have been confirmed in humans, while others, such as the relationship with metabolic disorders, are still under investigation. Some studies, which analyzed the biological role and mechanisms of tocopherols and tocotrienols over the past few years described new and even unexpected cellular and molecular properties that will be the subject of future research.

## 1. Introduction

Vitamin E is a group of eight compounds: α-, β-, γ-, δ-tocopherols and α-, β-, γ-, δ-tocotrienols, which are lipid-soluble [1,2]. Only photosynthetic organisms—plants, algae, and cyanobacteria as well as fungi, corals, sponges, and tunicates—have the ability to synthesize these compounds [2,3]. The main natural source of tocopherols and tocotrienols is the oily fraction of nuts and oil seeds [4,5]. Data on their presence in fruits and vegetables are often contradictory. This is due to the variety of analytical methods that can be used for their determination in food products [6]. The main sources of tocopherols are almond oil and other nut oils, olive oil, sunflower oil, rapeseed oil, corn oil, linseed oil, and soybean oil. Tocotrienols, in turn, can be found in palm and rice bran oil, wheat germ, barley, oats, hazelnuts, maize, and in annatto oil [4,7]. Habitual diet supplies mainly tocopherols, especially α-tocopherol and γ-tocopherol. γ-tocopherol is the most common in the US diet due to the higher consumption of soybeans, sesame, and corn oil, and α-tocopherol is the most common in the European diet [8]. In spite of the similar structure and antioxidant activity, vitamin E isoforms differ in bioavailability and metabolism [9]. All isoforms are biologically active, but only α-tocopherol is retained at high levels in plasma and tissues. The selectivity is achieved by the action of the hepatic α-tocopherol transfer protein (α-TTP) [10]. Plasma and body tissues are 90% saturated with α-tocopherol while other forms of vitamin E are degraded and excreted [11,12].

Except for the above-mentioned antioxidative activity of all vitamin E isoforms, antiproliferative, pro-apoptotic, anti-angiogenic, and anti-inflammatory effects are indicated. The beneficial impact on human health may also result from the ability to modulate signal transduction and gene expression in inflammation and immune system disorders [13,14]. 

Therefore, the aim of the present work is to describe known activities of both tocopherols and tocotrienols. This work focused on a review of the factors that influence the activity of compounds belonging to the vitamin E family, and their roles for human health are described. Based on the current scientific research, this paper presents the known and sought mechanisms of action of tocopherols and tocotrienols in the prevention of diet-related diseases, their differentiated bioactivity as well as the determinants of distribution and metabolism in humans.

## 2. Literature Search

The presented review shows current information on the known and sought-after properties of tocopherols, tocotrienols, and their metabolites as well as their importance in human health. The literature search was conducted between September 2020 and February 2021 in PubMed and ScienceDirect for articles related to vitamin E, using specific keywords such as tocopherol, tocotrienol, vitamin E, vitamin E metabolites, and health. Regarding health, the strongest emphasis was based on the most common and discussed disease entities, such as cardiovascular diseases, cancer, and obesity. Original articles on tocopherols and tocotrienols, published in English, were used. The review included clinical trials, controlled trials, cohort studies, systematic reviews, and meta-analyses.

## 3. Vitamin E Isoforms and Their Bioactivity

**Figure 1:**
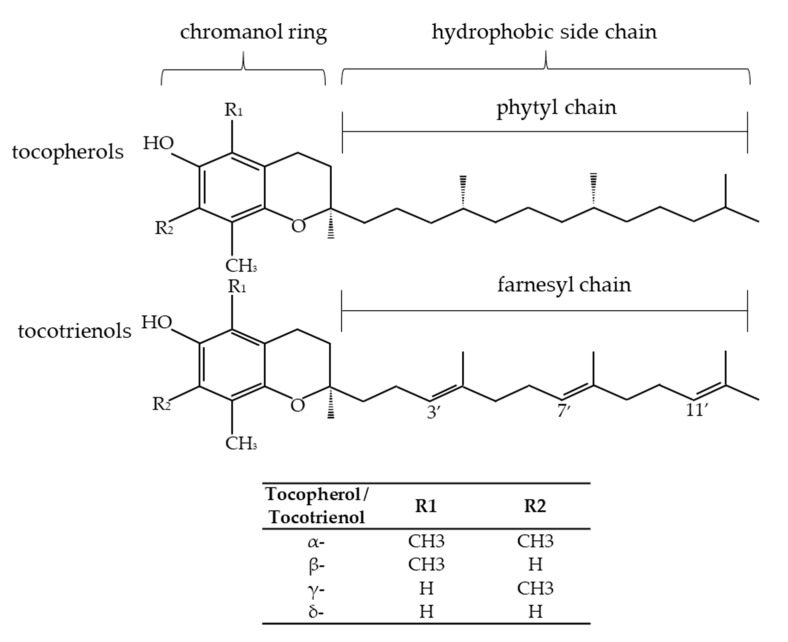
The structure of tocopherols, tocotrienols, and differences in the structure of their isoforms [17,18].

Tocochromanols, known as vitamin E, are the most common and dominant chromanols in the nature. Tocochromanols belong to a group of lipid-soluble antioxidants present in the plastids of plants [15]. Tocochromanols are a group of compounds synthesized only by plants and photosynthetic microorganisms. Most often eight compounds are mentioned as vitamin E: four tocopherols and four tocotrienols. In the group of tocotrienols, two further homologues occurring in rice bran have been identified. These are desmethylotocotrienol (d-P21-T3) and didesmethylotocotrienol (d-P23-T3), which differ from other tocotrienols by the lack of methyl groups in the benzene ring [16]. Tocochromanols are synthesized by plants from homogentisic acid [15]. Tocopherols and tocotrienols contain a chromanol ring (they are bicyclic phenols) and a hydrocarbon side chain. They consist of homologues α-, β-, γ-, δ- and differ in the number and location of the methyl substituent in the hydrophilic head of 6-chromanol, which is responsible for the presence of various isomeric forms of tocopherols and tocotrienols. Tocopherols are characterized by a saturated side chain (an aliphatic phytyl side chain), and tocotrienols have three double bonds in the side chain (an unsaturated farnesyl side chain) (Figure 1) [3].

Due to their structure, they are incorporated into the amphipathic phospholipid bilayer of cell membranes. Thereby, they are able to protect membrane lipids, organs, and organs of photosynthesis in plants from oxidative stress [19,20]. Each of the tocopherols has three chiral centers, at C2′, C4′, and C8′ carbon atoms, which can have the R or S configuration, resulting in eight possible stereoisomers. The naturally occurring form of α-tocopherol is RRR-α-tocopherol [21]. The chemically synthesized α-tocopherol is a racemic mixture of all possible α-tocopherol isomers in equimolar concentrations (synthetic form: all-racemic-α-tocopherol) [22]. Results of many studies established that the animal organisms (including humans) preferentially absorb the natural stereoisomer RRR-α-tocopherol [23] due to the affinity of α-tocopherol transfer protein (α-TTP) to forms of R [19]. Studies have shown that only natural α-tocopherol (RRR-α-tocopherol) is the most biologically active, and the activity of non-α-tocopherol forms is expressed as the α-tocopherol equivalent (%), which accounts for 50% for β-tocopherol, 10% for γ-tocopherol, 3% for δ-tocopherol, 30% for α-tocotrienol, 8% for γ-tocotrienol, and 5% for β-tocotrienol. For δ-tocotrienol, the equivalent has not been established [24]. According to the Food and Nutrition Board of the Institute of Medicine, only α-tocopherol is recognized as a nutrient (vitamin) that is able to meet vitamin E requirements in humans. Additionally, the differences in antioxidant activities of vitamin E forms are relatively minor, while their biological activities are divergent and multidirectional [25]. 

## 4. Absorption, Bioavailability, and Biotransformation of Tocopherols and Tocotrienols

The amount of vitamin E absorbed depends on the differences in the food matrix that supplies this vitamin. It is known that retinoic acid, plant sterols, eicosapentaenoic acid, alcohol, and dietary fiber inhibit the absorption of vitamin E [26]. Studies have shown low bioavailability of vitamin E isoforms from the apples matrix and high bioavailability from the bananas, bread, and lettuce matrix. Additionally, they were more bioavailable from egg-free durum wheat pasta than from pasta containing eggs. The fragmentation of the food matrix may increase the bioavailability of vitamin E isoforms and their transfer to micelles; no similar effect was found for technological treatment (either thermal or high-pressure treatments). The higher the amount of fat in a meal, the higher absorption of vitamin E compounds occurs [13]. In the gastrointestinal tract, tocopherols and tocotrienols are absorbed to a similar extent, but their absorption depends on adequate pancreatic function, bile secretion, and the formation of micelles [13]. It turned out that transmembrane proteins play a key role in the intestinal absorption of vitamin E [27]. Initially, it was thought that vitamin E absorption occurred by passive diffusion through the enterocyte membrane [13]. In later years, it was shown that absorption is also mediated, at least in part, by three groups of proteins: Niemann–Pick C1-like 1 protein (NPC1L1), scavenger receptor class B type 1 (SRB1), and a cluster of determinant 36 (CD36). These three proteins are mainly described as cholesterol transporters, but they can also bind to the other substrates [20,28]. The absorption of tocopherols and tocotrienols in the intestine varies from 20% to 80% of the total ingested amount and is lower than the other fat-soluble vitamins [29]. The concentration of tocopherols and tocotrienols in the plasma is influenced by their content in a diet, absorption, and their metabolism. Some of these factors may be modulated by emerging genetic changes in the genes, which encode proteins responsible for the above factors [30]. 

**Figure 2:**
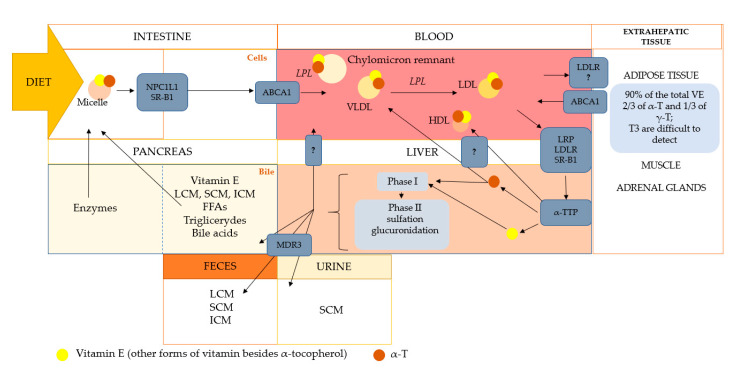
The simplified scheme of the transport and metabolism of vitamin E. The metabolism of vitamin E follows generally like other lipids species. In the intestine, vitamin E, along with other lipids, is packed into micelles, which are captured by receptors. In the intestinal epithelial cells, vitamin E is incorporated into chylomicrons or HDL via ABCA1. Vitamin E in the blood follows the lipoprotein transport route and is delivered to the liver or extrahepatic tissues. Vitamin E transport takes place with the participation of chylomicrons, which are subjected to hydrolysis by lipoprotein lipase. Further transport of vitamin E is through by chylomicron remnants, HDL, LDL, and VLDL. In the liver, vitamin E is sorted and directed to catabolism or to various lipoproteins (the mechanisms are not fully understood), returning to the bloodstream. The transport route is the same for all forms of vitamin E. Discrimination of the other forms in favor of α-tocopherol occurs in the liver by α-TTP, which protects against excessive degradation and excretion of α-tocopherol. The remaining forms of vitamin E are included in catabolism (phase I and II). NPC1L1, Niemann-Pick C1 Like 1 protein; SR-B1, scavenger receptor class B type 1; ABCA1, ATP-binding cassette transporter; VLDL, very-low-density lipoproteins; HDL, high-density lipoproteins; LDL, low-density lipoproteins; LDLR, LDL receptor; VE, vitamin E; α-T, alpha-tocopherol; γ-T, gamma-tocopherol; T3, tocotrienols; LRP, LDL receptor-related protein; LCM, long-chain metabolites (13′-COOH); ICM, intermediate-chain metabolites (11′-COOH, 9′-COOH); SCM, short-chain metabolites (7′-COOH, 5′-COOH, 3′-COOH); FFAs, free fatty acids; MDR3, multidrug resistance protein 3. The figure was modified from [1,32,33,34,35,36].

Vitamin E is transported in the blood by plasma lipoproteins and erythrocytes. After entering the circulation, chylomicrons undergo a reconstruction process consisting mainly of the hydrolysis of triglycerides by lipoprotein lipase, resulting in the formation of chylomicron residues. Part of vitamin E forms is taken up by extrahepatic tissue, and the remaining part of vitamin E incorporated in the chylomicron remnants is taken up by the liver [30]. Due to the presence of protein α-TTP in the liver, α-tocopherol is preferentially transported further, while the remaining tocochromanols are metabolized and excreted in the bile. α-TTP mediates the incorporation of α-tocopherol into very-low-density lipoproteins (VLDL) and the secretion of these complexes into circulation. In the blood, VLDLs are catabolized to low (LDL) and high-density (HDL) lipoproteins by lipoprotein lipase (LPL). VLDL catabolism causes α-tocopherol to occur simultaneously in all the above-mentioned types of lipoproteins. α-tocopherol that is delivered into LDL lipoproteins is transferred to the tissues, where it performs its functions (Figure 2) [31].

The uptake of different forms of vitamin E into the liver is probably nonspecific. The mechanisms that are involved are promiscuous in that these are general xenobiotic processes [37,38]. It is worth noticing that α-TTP has 100% affinity for α-tocopherol, 38% for β-tocopherol, 9% for γ-tocopherol, and 2% for δ-tocopherol [1]. Research results confirmed the existence of bio-discrimination against tocotrienols due to the affinity of tocopherols to the α-TTP protein [39]. Among the tocotrienols, the α- form is known for the highest oral bioavailability. A low level or lack of α-tocopherol, as well as the higher content of α-tocotrienol in the food matrix are the determinants of increased absorption of the ingested tocotrienols [29]. Due to their low affinities for hepatic α-TTP, non-α-tocopherol forms are less efficiently transferred to VLDL and are detected in the blood and tissues in low concentrations [37]. Both tocopherols and tocotrienols are accumulated in many tissues, among others, the liver, adrenal glands, and adipose tissue [40]. It is estimated that 90% of the total amount of vitamin E is accumulated in the adipose tissue, mainly in adipocyte lipid droplets [32]. Vitamin E that is accumulated in adipose tissue consists of about two-thirds of α-tocopherol and one-third of γ-tocopherol [33]. Accumulated tocotrienols are difficult to detect, but supplementation of tocotrienols in animals increased their pool in the adipose tissue [34]. With a vitamin E-free diet applied for four weeks in rats, a decrease of tocopherol and tocotrienol was observed but only in the liver, not in the adipose tissue [39]. Supplementation of α-tocopherol and tocotrienols significantly increased the level of α-tocopherol in the plasma, reaching a maximum concentration 8 h after supplementation and maintaining a high level even after 24 h. Tocotrienols reached their maximum concentration 4 h after supplementation. Then their level decreased significantly, and they completely disappeared from the plasma after 24 h [35]. Moreover, accumulation of α-tocotrienol in selected organs was observed in rats after supplementation with α-tocotrienol (5 mg/kg body weight) for over two years. When vitamin E-deficient diet was applicated, the accumulated α-tocotrienol was depleted after less than two months, while the loss of α-tocopherol was negligible [41]. 

Human studies also provide evidence for α-tocopherol retention and degradation of other non-α-tocopherol forms. Uchida et al. [42] even noted that γ-tocopherol was metabolized and excreted faster than α-tocopherol. Additionally, faster turnover of tocotrienols was seen in humans [43]. The action of non-α-tocopherol homologues in the human body is limited because of the fact that they are immediately metabolized in the liver and excreted in bile or urine. Recent studies have highlighted that tocotrienols exhibit higher antioxidant activity in in vivo systems. However, their oral bioavailability is significantly limited as they are not recognized by α-TTP. Additionally, their occurrence in food is low or even rare [44]. Due to their fast metabolism their lifetime is short. They quickly penetrate the skin, combating the effects of oxidative stress induced by UV radiation and ozone [16]. Non-α-tocopherol forms are recognized as xenobiotics and are metabolized and excreted. Consequently, their plasma concentrations are decreased, and formed metabolites concentrations are increased [43]. Tocotrienols metabolites formed in the liver are the promising forms of vitamin E in the prevention of diseases caused by acute inflammation and oxidative processes [44].

Recent studies have found that supplementation with a mixture of vitamin E isoforms resulted in a significant increase in the concentration of tocotrienols in the tissues. This may indicate a different mechanism of intracellular transport of α-tocotrienol, independent of α-TTP. Each cell type likely has a different selectivity for the tocotrienols uptake, e.g., it was found that sirtuin 1 (SIRT 1) proteins in human fibroblasts may regulate the uptake and bioavailability of tocotrienol isomers [40]. Additionally, other proteins—tocopherol-associated proteins 1, 2, 3 (TAP 1, 2, 3), human plasma protein afamin, albumin, and phospholipid transfer protein (PLTP)—show binding capacity to other forms of vitamin E [45]. An alternative explanation for the metabolism of other forms of vitamin E was proposed after identifying the metabolites of vitamin E—carboxyethyl-hydroxychroman (CEHC)—and discovering that this pathway preferentially metabolizes non-α-tocopherol forms in hepatic cells [1]. Vitamin E isoforms that are not transferred from the liver by α-TTP are metabolized in the phase I (catabolism and side-chain shortening) and phase II (sulfation and glucuronidation) [46]. The catabolism of α-tocopherol and other isoforms occurs when the amount of hepatic α-tocopherol exceeds α-TTP’s transfer capacity. α-Tocopherol is endogenously converted to α-CEHC (2,5,7,8-tetramethyl-2-(2′-carboxyethyl)-6-hydroxychroman). Urinary excretion of α-CEHC positively correlates with α-tocopherol increase in both diet and plasma concentration in healthy subjects [47]. It is not clear whether the increase in urinary α-CEHC excretion is related to the increased consumption of α-tocopherol in a single meal or whether these changes reflect long-term higher consumption of this nutrient [38,47]. 

Initially, it was believed that the metabolism of α-tocopherol occurs through the opening of the chromanol ring and the subsequent degradation of the side chain, when only two metabolites are produced—namely, α-tocopheronic acid and its lactone, α-tocopheronolactone (α-TL), so-called Simon’s metabolites [48,49]. In later years, additional metabolic pathways were defined following the discovery of other α-tocopherol metabolites with an intact chromanol ring. ω-Hydroxylation of the aliphatic side chain leads to the formation of 13′-hydroxychromanol (13′-OH), and 13′-carboxychromanols (13′-COOH) are formed as a result of oxidation. The subsequent stages of oxidation lead to shortening of the side chain, creating further metabolites—carboxydimethyldecylhydroxychromanol (CDMDHC, 11′-COOH), carboxymethyloctylhydroxychromanol (CDMOHC, 9′-COOH), carboxymethylhexylhydroxychromanol (CDMHHC, 7′-COOH), and carboxymethylbutylhydroxyxhromanol (CMBHC, 5′-COOH). The end products of vitamin E metabolism are carboxyethyl-hydroxychromanols (CEHC), referred to as 3′-COOH or short-chain metabolites (SCM). Research confirms that tocotrienols follow the same metabolic pathway [26]. 

Non-α-tocopherol forms are preferentially catabolized in the oxidation of side chains with the formation of hydroxylated and carboxylated chromanols [50]. Vitamin E isoforms metabolism includes the phase I ω-hydroxylation to the alcohol derivative of 13′-OH, catalyzed by cytochrome P450 monooxygenase (CYP4F2), which takes place in the endoplasmic reticulum of liver cells. These products are considered as a phase I metabolic intermediate and limit the accumulation of the lipophylic vitamin [34,43]. The oxidized hydroxyl group leads to the formation of 13′-COOH long-chain metabolites (LCMs) under the action of aldehyde dehydrogenase. It is followed by a series of β-oxidations with the formation of medium and short-chain carboxychromanols—11′-COOH, 9′-COOH (in peroxisomes) and 7′-COOH, 5′-COOH (in the mitochondrial matrix)—and the final metabolite, 3′-COOH (CEHC). The metabolites are excreted from the body with urine and feces [46]. Metabolites occur in human urine in free form or as sulfates or glucuronides [51,52]. Because of the degradation of tocotrienols, CEHCs are formed, which suggests a similar metabolic mechanism as in tocopherols. The side-chain double bonds undergo a saturation step catalyzed by 2,4-dienoyl-CoA reductase and 3,2-enoyl-CoA isomerase (coenzyme A). They are also involved in the metabolism of unsaturated fatty acids [2]. 

## 5. The Bioactivity of Vitamin E Metabolites

Recent studies suggest that the properties of LCMs of vitamin E correspond to the functions of vitamins A and D. The structural similarity of vitamin E metabolites with vitamin A and D metabolites (9-cis-retinoic acid and 1,25(OH)2D3) allows us to reach the conclusion that there are also vitamin E-specific receptors, hitherto undiscovered. This hypothesis is likely confirmed by the findings on the regulatory activities of vitamin E metabolites. Although plasma concentrations of the metabolites are low, the results of animal and in vitro studies have indicated their strong biological potential [51]. Studies have reported that the functions of vitamin E metabolites can be divided in terms of anti-inflammatory activity, antitumor activity, participation in the regulation of cellular lipid homeostasis, drug interactions, and regulation of the metabolites’ own metabolism [51,53]. 

Research on anti-inflammation activity often focuses on the possibility of regulating the pro-inflammatory enzymes by vitamin E metabolites [54,55]. Cyclooxygenase (COX) that catalyzes pro-inflammatory eicosanoid production plays an important role in regulation of the inflammatory response and contributes to the development of many chronic diseases, such as cancers. The inhibitory effect of α-tocopherol metabolites—α-9′-COOH and α-13′-COOH—on the activity of COX-1 and COX-2, catalyzing the production of pro-inflammatory eicosanoids, was demonstrated [56]. Studies have shown that α-13′-COOH is a competitive COX inhibitor and therefore competes to join to the substrate-binding site. It shows a greater affinity for cyclooxygenases than other metabolites and forms of tocopherols [54].

The α-tocopherol metabolite α-13′-COOH inhibits inflammation by targeting 5-lipoxygenase (5-LO), which catalyzes the initial stages of biosynthesis of strong immunomodulatory lipid mediators [57,58]. Leukotrienes, generated by the enzyme 5-LO through metabolism, play a major role in the development of asthma and allergic rhinitis. They also contribute to oxidative DNA damage and consequently to cardiovascular diseases, inflammatory liver diseases, neurodegenerative disorders, and cancers [59,60,61]. It has been shown that the α-13′-COOH is accumulated at sites of inflammation and suppresses acute inflammation in murine peritonitis [57]. 

Studies focused on antitumor properties have shown that α- and δ-tocopherol metabolites stopped proliferation in the human hepatocyte HepG2 cancer cell line [62]. Both metabolites α-13′-COOH and δ-13′-COOH effectively inhibited cell growth while no such effect was noticed among the forms of hydroxy metabolites [53,62].

Additionally, Jang et al. [63] found a similar effect for 13′-COOH, derived from δ-tocopherol and δ-tocotrienol. In a mice model of colon cancer, they inhibited COX-2 and 5-LO and thus induced apoptosis and autophagy. They are seemingly promising factors that may act against cancer.

The metabolite of γ-CEHC, unlike α-CEHC, is attributed to natriuretic properties, including the regulation of the water–sodium balance and maintenance of cardiovascular homeostasis. Both γ-tocopherol and γ-CEHC also inhibit the activity of COX-2 [16]. 

Many of the studies have focused on vitamin E metabolites and their influence on the regulation of key pathways in the development of macrophage foam cells [64,65]. Macrophages bind oxidized LDL cholesterol (oxLDL) through many types of receptors, among them scavenger receptor CD36, which is also involved in the transport of tocopherol (as well as a number of lipid compounds) [8]. The expression of CD36 is reduced by α-tocopherol [65], while long-chain α-tocopherol metabolites (α-13′-OH and α-13′-COOH) influence oxLDL uptake independent of CD36 [64]. They can inhibit the formation of macrophage foam cells, thus having a positive effect on the prevention of atherosclerosis [64]. 

Torquato et al. [66], found that the α-13′-OH metabolite increased the expression of the CYP4F2 protein gene in human hepatic HepG2 cells. This protein is involved in the metabolism of vitamin E. This proves that derivatives of vitamin E metabolism, mainly α-13′-OH, may be responsible for the existence of a positive regulatory feedback loop that occurs in the metabolism of vitamin E.

The results of the research also indicate that α-13′-COOH and γ-tocotrienols activated the pregnane X receptor (PXR), which is a transcription factor of P-glycoprotein (P-gp), which is responsible for the intracellular concentration and transport of pharmaceuticals. What is more, it induced protein expression and P-gp transporter activity [67]. This means that in patients receiving γ-tocotrienol supplementation or high doses of α-tocopherol, drug levels that are P-gp substrates should be monitored.

## 6. Proven Antioxidant and Anti-Inflammatory Effects of α- and γ-Tocopherols, and What Is the Role of Other Isoforms?

α-Tocopherol is a specific non-enzymatic, chain-breaking antioxidant in aerobic organisms. It is present in cell membranes and plays a significant preventive role in the oxidative damage of molecules such as DNA or lipids [20,68]. α-Tocopherol neutralizes free radicals and breaks the chain reaction in the oxidation of the polyunsaturated fatty acids [69]. This activity is associated with the non-esterified hydroxyl groups of the chromanol ring. Moreover, the lipophilic tail of tocopherol can interact with cellular lipids and other molecules, protecting them from oxidation or peroxidation [8]. The α-tocopheroxyl radical is relatively long-lived, and it can be reduced to the α-tocopherol by water-soluble antioxidants such as ascorbic acid [43].

It is reported that the antioxidant activity of vitamin E isomers depends on the number of hydroxyl groups and is in the order of α > β > γ > δ [21]. Some research results indicate a greater antioxidant potential of tocotrienols than of tocopherols [70]. This may be due to their greater distribution in the phospholipid bilayer of cell membranes and more effective interaction with lipid peroxyl radicals [71]. However, compared to tocopherols they are less orally bioavailable [72]. Moreover, when they become radicals, they are more reactive and can readily form adducts that are potentially cytotoxic [43].

In addition, other forms of vitamin E, for instance, γ-tocopherol, δ-tocopherol, and γ-tocotrienol, have unique antioxidant properties. It has been observed that γ-tocopherol has the ability to scavenge reactive forms of nitrogen. This activity is not observed for α-tocopherol [50]. 

Moreover, at the molecular level, non-antioxidative functions of tocopherols and tocotrienols have been shown [45]. This activity is possible because of specific interactions with enzymes, structural proteins, structural lipids, and transcription factors [73]. The main effect of α-tocopherol is the inhibition of the activity of protein kinase C (PKC). It affects the proliferation of monocytes, macrophages, and neutrophils of smooth muscle cells and reduces the production of superoxide free radicals in neutrophils and macrophages. α-Tocopherols can modulate of phospholipase A2 activity and inhibition of prostaglandin activity E2 and cyclooxygenase 2. In the regulation of gene expression, several genes have been described as modulated by tocopherol. Several possible regulatory pathways have been described for α-tocopherol, which can alter the activity of transcription factors and induction pathways through enzyme modulation and may ultimately affect gene expression. Vitamin E isoforms can directly modulate the activity of transcription factors through pregnane X receptor (PXR), peroxisome proliferator-activated receptors (PPARs), orphan nuclear receptors, or one of the three human α-tocopherol associated proteins (hTAPs). Additionally, they can affect gene expression by binding to human α-tocopherol-related hTAP proteins, which regulate tocopherol access to specific enzymes and transcription factors and control the level of “free” tocopherol. Ultimately, tocopherols and tocotrienols can be metabolized to bioactive compounds (metabolites) and affect the activity of transcription factors [71,74].

Both the anti-inflammatory and antioxidant functions of vitamin E may enhance the immune system [75]. In addition, vitamin E regulates the maturation and functioning of dendritic cells, whose role is to connect the innate and adaptive immune system to coordinate the immune response. Other main roles of vitamin E isoforms include the increase in NK cells (natural killer cells) activity, humoral response, antibody function, and the improvement of T lymphocyte synapses formation as well as the initiation of the T cell activation signal [76,77,78]. Later studies revealed that various forms of vitamin E act as signaling and gene-regulating molecules and indicated a nonantioxidant molecular function of α-tocopherol [79]. However, Traber and Atkinson [74] underlined that the mechanism of action of α-tocopherol results from its antioxidative role. It seems that this fundamental role of compounds with vitamin E activity is unquestionable, while its other properties require confirmation in further in vivo research.

Interest in tocopherols and tocotrienols has grown in recent years due to the emerging evidence that they can prevent common diseases. Both tocopherols and tocotrienols have been shown as compounds with the following properties: anti-atherosclerotic [80], anti-cancer [42,50,81,82], anti-allergic [83], improvement of immune functions [75], anti-cardiovascular disease [84], anti-lipidemic [85], anti-diabetic [86], antihypertensive [87], anti-inflammatory [88], anti-obesity [5], and anti-non-alcoholic steatohepatitis (NASH) [89]. On the other hand, the results of many clinical trials do not confirm the protective role of α-tocopherol in preventing disease in people with adequate nutritional status. Based on the current state of research, γ-tocopherol and tocotrienols, as well as metabolites of α- and γ-tocotrienol, are promising compounds in the prevention of diseases driven by acute inflammatory and oxidative damage. To verify their biological role, large-scale clinical trials are needed [44].

## 7. α-Tocopherol Status and Requirements

The assessment of the human requirement for α-tocopherol is hampered by the rare occurrence of clinical symptoms of deficiency [3]. Symptoms usually develop in premature babies, infants, and adults with fat malabsorption, liver disease, or genetic diseases [78]. Extremely low values of α-tocopherol in the body may lead to disease named ataxia with vitamin E deficiency (AVED). This is a rare disorder caused by a mutation in the gene encoding α-TTP. Patients with AVED have the ability to absorb vitamin E isoforms in the intestine, but they have extremely poor ability to retain it. Symptoms of the disease include as follows: progressive ataxia, clumsiness of the hands, loss of proprioception, areflexia, retinal atrophy, degeneration of the spine, accumulation of lipofuscin in neurons, and loss of Purkinje cells. The disease causes low blood levels of α-tocopherol, but it can be prevented by α-tocopherol supplementation. People who suffer from α-tocopherol deficiency because of genetic mutations also have impaired selectivity between α-tocopherol and γ-tocopherol, and increased excretion of α-CEHC [45,90]. Other forms of vitamin E than α-tocopherol are not effective against the human deficiency disease [91]. Severe α-tocopherol deficiency causes significant neuronal disorders, such as ataxia and oxidative disorders, cardiovascular diseases, cancer, and cataracts. As pointed out by Azzi [91], only α-tocopherol should be called vitamin E, so consistently, both in the diet and in recommendations, only this form of the vitamin E family should be considered.

Now, 8–15 mg of α-tocopherol or an α-tocopherol equivalent for women and men, according to the different scientific institutions, is recommended [25,92,93,94]. There are significant differences in α-tocopherol intake in different countries, varying from 8 to 10 mg/person/day in Finland, Iceland, Japan, and New Zealand, to 20 to 25 mg/person/day in France, Greece and Spain [3]. However, in a study that focused on a comparison of vitamin E intake in different subpopulations, over 80% of the mean and the median data points were below the RDA of 15 mg/day [95].

It was also noted that the requirements for α-tocopherol depend on the content of polyunsaturated fatty acids (PUFA) in a diet [96]. Peroxyl radicals react 1000 times faster with α-tocopherol than with polyunsaturated fatty acids (PUFA), which prevent their further oxidation [97]. Therefore, to quantify the need for α-tocopherol, in addition to the basal need, additional amounts depending on PUFA intake should be considered. It has been estimated that the need for α-tocopherol should be in the range of 12–20 mg/day for the “typical” range of PUFA consumption [96].

The concentration of α-tocopherol in serum or plasma is the main method used for α-tocopherol status assessment. According to Traber et al. [98], levels of α-tocopherol in serum below 9 µmol/L in men and below 12 µmol/L in women are considered as deficiency. A large prospective cohort analysis with more than 30 years of follow-up has provided strong evidence that men with higher serum α-tocopherol levels (≥14.2 mg/L vs. <9.3 mg/L) had lower overall mortality and lower mortality from cardiovascular disease (CVD), heart disease, stroke, cancer, and respiratory disease [99]. In cancer prevention studies, higher blood levels of α-tocopherol were associated with lower mortality. The lowest total mortality was observed at the concentration of α-tocopherol at the level of 30 µmol/L in the blood serum [100,101]. Additionally, other results of observational studies showed that at the point of 30 µmol/L and above, the concentration of α-tocopherol in the serum has a positive effect on human health [102]. However, as it was summarized by Eggersdorfer [95], only 21% of the reported populations and subpopulations reached this threshold, which may indicate a generally low vitamin E (α-tocopherol) nutritional status worldwide. No reference values have been established for the other forms of vitamin E. According to Traber [43], plasma α-tocopherol concentration is not a reliable marker for the assessment of vitamin E status, especially in subjects with an abnormal lipids profile. Therefore, adjusting α-tocopherol to plasma lipids and lipoproteins is recommended. However, it is not widely used, and it may be a cause of overinterpretation and ambiguity of the described results about α-tocopherol status. Plasma α-tocopherol concentration may be modified by several factors such as age, gender, lifestyle, low circulating lipid levels, genetic variation and variation in the absorption, metabolism, and excretion of vitamin E, as well as by obesity, metabolic syndrome, or high levels of oxidative stress [5,12]. Urine or plasma α-CEHC has been suggested as a better biomarker of adequate vitamin E status, but the methodology was not sensitive enough to detect their low levels and thus they are not widely used [43]. Additionally, the assessment of vitamin E status is also difficult because it is a fat-soluble vitamin, which is stored in adipose tissue [12]. 

It is generally accepted that the concentration of α-tocopherol in adipose tissue reflects the long-term concentration of vitamin E [12]. Studies suggest that the α-tocopherol that is stored in the tissues would not be released on demand, but its concentration depends on the lipid content of the tissue. Adipose tissues were used to assess the long-term status of α-tocopherol, and it was determined that the typical concentration of α-tocopherol in adipose tissue in adults is about 100–300 µg/g (200–700 nmol/g). Additionally, the concentration of α-tocopherol in adipose tissue was used to determine the adequacy of vitamin E supplementation in patients with deficiency and was related to the concentration in peripheral nerves [12]. Measuring this biomarker of vitamin E status requires a biopsy of adipose tissues that is not widely accepted and used.

In conclusion, validated questionnaires and appropriate biomarkers are needed to assess vitamin E intake and status. To assess the dietary intake, mainly dietary records are used; however, they may lead to losing the key sources of vitamin E such as nuts and fats. In turn, for a nutritional status assessment, an adjustment for plasma lipids should be applied. If possible, the concentration of metabolites in the plasma and urine should be measured.

## 8. Adiposity and Vitamin E status

The existing results indicated a significant relationship between the content of adipose tissue in the body and the demand and metabolism of tocopherols and tocotrienols, mainly α-tocopherol. An excessive level of adipose tissue generates chronic inflammation visible in the increased secretion of cytokines, proteins, and immune response mediators, leading to the activation of inflammatory signaling pathways. Chronic, low-grade inflammation in obesity leads to increased oxidative stress and a disturbed balance of oxidants and antioxidants in the body [103]. Bioactive dietary antioxidants such as tocopherols and tocotrienols can prevent damage caused by inflammation and reactive oxygen species, thereby reducing the negative effects of obesity. On the other hand, an excess of adipose tissue may generate in the body an increased demand for antioxidants, which may cause their greater utilization, leading to their decreased concentration in the blood [104]. 

Research into the relationship between obesity and plasma α-tocopherol concentration is inconclusive. In some studies, the concentration of tocopherols (α-, γ-) in blood plasma was significantly and positively associated with anthropometric indices, the strongest associations of which were with waist-to-hip ratio (WHR), body mass index (BMI), waist circumference (WC), waist-to-height ratio (WHtR), indicative of central obesity [104,105]. Some studies have found opposite results and a negative relationship between plasma α-tocopherol concentration and the incidence of central obesity [106,107,108]. The results of Barzegar-Amini and colleagues’ studies [68] showed that low serum α-tocopherol levels were significantly associated with increased waist and hip circumference, body weight, and cholesterol and triglyceride levels. It can be assumed that all these associations show certain tendencies, but the clinical significance of the associations may be questionable.

More valuable for further research seem to be the results concerning the relationships between adiposity and plasma α-tocopherol, other vitamin E isomers, or their metabolites concentration. Chai et al. [109] found significantly higher levels of γ-tocopherol in the blood in people with obesity compared with subjects with normal body weight (classification based on BMI) and a lack of differences between the groups for α-tocopherol [109]. It has also been shown that in people with metabolic syndrome, which was associated, i.e., with excessive adipose tissue, the level of excreted α-tocopherol metabolites as well as the plasma level of α-tocopherol were lower, and the level of oxidative stress increased [110].

In patients with excess body weight who remained on a reduction diet for six weeks, significant reduction in blood plasma α-tocopherol was observed. Almost 80% of them had this level below 20 μmol/L. This may indicate an increased risk for cardiovascular diseases and low antioxidant protection [103]. Similar results were obtained after the eight-week weight reduction program [111]. Eighty-five subjects with initially normal levels of α-tocopherol (mean 28 μmol/L) reduced their body weight by an average of 13%, but a significant decrease in the level of α-tocopherol (to about 21 µmol/L) was observed [111]. It is unclear whether the above-mentioned differences in distribution of the α-tocopherol and other isoforms are due to insufficient intake, changes in metabolism in obese individuals, or due to these two factors simultaneously [112]. This is explained by decreased α-tocopherol catabolism (by slower turnover) in people with excess body weight, although it is unclear whether this is due to greater oxidative damage, decreased dietary vitamin intake, or impaired absorption. Any reduction in the absorption of α-tocopherol limits its availability. In addition, inflammation and increased oxidation associated with metabolic disorders reduce the bioavailability of tocopherol [37].

Recently, the research results also indicated that tocotrienols may have a positive effect in reducing obesity. It has been shown that tocotrioenols inhibit adipogenesis by reducing the accumulation of triglycerides (TGs) and lipid droplets in murine Hepa 1-6 liver cancer cells, HepG2 human liver cancer cells, 3T3-L1 preadipocytes, and human adipose-derived stem cells (hASCs). γ-T3 was characterized by the greatest potency of the anti-adipogenic effect, followed by δ-, β-, and α- forms. Additionally, studies have reported that tocotrienols reduce body weight, especially fat mass in obese animals [112]. Uto-Kondo et al. [113] assessed the effect of a tocotrienol-rich fraction from palm oil on adipocyte differentiation in 3T3-L1 cells. The authors suggested that the α- and γ-tocotrienol fractions inhibited the differentiation of pre-adipocytes into adipocytes, potentially preventing obesity. A study in rats [114] showed that γ-tocotrienol (60 mg/kg body weight/day) decreased adipose tissue mass induced by various doses of glucocorticoid. 

Based on those results, some authors indicate that strong epidemiological evidence is still needed regarding the relationship between vitamin E intake, status, and weight loss in humans [115]. However, on the other hand, given tocopherols and tocotrienols metabolism and the existence of the specific proteins for α-tocopherol, it may be supposed that vitamin E, despite its undoubted bioactivity, will be not a remedy for metabolic syndrome or obesity. 

These results rather indicate the need for further research aimed at establishing the vitamin E requirements and desirable plasma concentrations for non-α-tocopherol forms for individuals with disorders associated with increased oxidative stress or inflammation [37,110]. Nonetheless, it is worth noting that people with excess body weight do not meet the requirements for many vitamins and minerals in their diet (the phenomenon of malnutrition in obesity), including vitamin E. It is often related to the reduction of fat in the diet, which is the main source of many lipid-soluble compounds, such as vitamin E [116]. 

## 9. Summary

**Figure 3:**
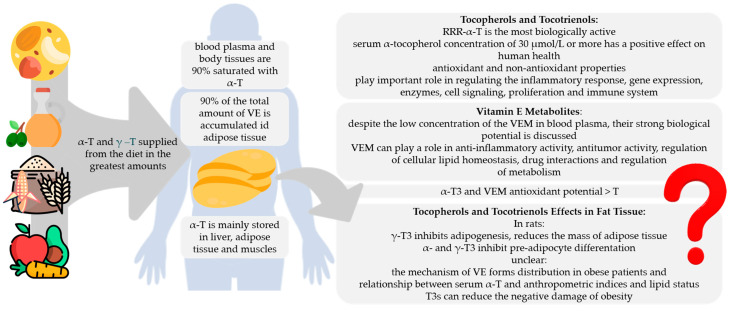
Summary of potential health benefits of tocopherols and tocotrienols. T, tocopherol; T3, tocotrienol; VE, vitamin E; VEM, vitamin E metabolites. The author’s own study based on [4,8,10,11,12,35,36,37,74,99,104,105,106,107,112,113,114]; icons source: www.flaticon.com (accessed on 31 March 2021).

This review presents the available evidence confirming the role of γ- and δ-tocopherol as well as α- and γ-tocotrienol as bioactive compounds that are defined to have documented health benefits beyond the normal nutritional effects and a positive effect on specific functions of the human body. The positive role of α-, γ-, and δ- tocopherol as well as α- and γ-tocotrienol and also their metabolites in cell homeostasis; modulation of signaling pathways, enzymes, and genes; and the mechanism of antioxidant action contributing to the reduction of inflammation was clearly documented. This activity is possible by specific interactions with enzymes, structural proteins, and structural lipids as well as by transcription. The metabolism of vitamin E is under constant control of the organism, and RRR-α-tocopherol as the most biologically active form is preferred. Although many studies confirm the multidirectional activity of vitamin E isoforms, the exact mechanisms require further research. In addition to its influence on fertility, early animal studies documented that this vitamin is a necessary factor for other vital functions and for the development of tissues and organs such as brain and nerves, muscles and bones, skin, bone marrow, and blood. Some of these functions have been confirmed in humans, while others are still under investigation. Over past few years, several laboratories have described new and even unexpected cellular and molecular properties of tocopherols, tocotrienols, and their metabolites (Figure 3).

In many scientific studies, the terms α-tocopherol and vitamin E are used interchangeably, which can result in ambiguity and confusion. The need to specify nomenclature, to stop combining isoforms into one vitamin E compound, is highlighted. This is mainly due to the differences between the various forms of vitamin E as well as the differences in the mechanisms of action and biological activity between tocopherols and tocotrienols as well as the fact that α-tocopherol is the form of vitamin E that preferentially accumulates in blood and tissues This aspect, similar to the consideration of lipids/lipoproteins in the determination of plasma tocopherols levels, needs to be systematized. We believe that this updated knowledge is worth considering as a way of improving the nutritional recommendations and for creating the criteria for a new wave of research into tocopherols and tocotrienols and their relationship with health.

## Data Availability

Not applicable.

## References

[1] Galli F., Azzi A., Birringer M., Cook-Mills J.M., Eggersdorfer M., Frank J., Cruciani G., Lorkowski S., Özer N.K. (2017). Vitamin E: Emerging aspects and new directions. Free Radic. Biol. Med..

[2] Birringer M., Siems K., Maxones A., Frank J., Lorkowski S. (2018). Natural 6-hydroxy-chromanols and -chromenols: Structural diversity, biosynthetic pathways and health implications. RSC Adv..

[3] Food and Agriculture Organization, World Health Organization (1998). Vitamin and Mineral Requirements in Human Nutrition.

[4] Shahidi F., De Camargo A.C. (2016). Tocopherols and tocotrienols in common and emerging dietary sources: Occurrence, applications, and health benefits. Int. J. Mol. Sci..

[5] Waniek S., di Giuseppe R., Plachta-Danielzik S., Ratjen I., Jacobs G., Koch M., Borggrefe J., Both M., Müller H.-P., Kassubek J. (2017). Association of vitamin E levels with metabolic syndrome, and MRI-derived body fat volumes and liver fat content. Nutrients.

[6] Knecht K., Sandfuchs K., Kulling S.E., Bunzel D. (2015). Tocopherol and tocotrienol analysis in raw and cooked vegetables: A validated method with emphasis on sample preparation. Food Chem..

[7] Pandya J.K., DeBonee M., Corradini M.G., Camire M.E., McClements D.J., Kinchla A.J. (2019). Development of vitamin E-enriched functional foods: Stability of tocotrienols in food systems. Int. J. Food Sci. Technol..

[8] Galmés S., Serra F., Palou A. (2018). Vitamin E Metabolic Effects and Genetic Variants: A Challenge for Precision Nutrition in Obesity and Associated Disturbances. Nutrients.

[9] Jiang Q. (2017). Natural Forms of Vitamin E as Effective Agents for Cancer Prevention and Therapy. Adv. Nutr..

[10] Hosomi A., Arita M., Sato Y., Kiyose C., Ueda T., Igarashi O., Arai H., Inoue K. (1997). Affinity for α-tocopherol transfer protein as a determinant of the biological activities of vitamin E analogs. FEBS Lett..

[11] Lee P., Ulatowski L.M. (2019). Vitamin E: Mechanism of transport and regulation in the CNS. IUBMB Life.

[12] Traber M.G., Leonard S.W., Traber D.L., Traber L.D., Gallagher J., Bobe G., Jeschke M.G., Finnerty C.C., Herndon D. (2010). α-Tocopherol adipose tissue stores are depleted after burn injury in pediatric patients. Am. J. Clin. Nutr..

[13] Reboul E. (2017). Vitamin E bioavailability: Mechanisms of intestinal absorption in the spotlight. Antioxidants.

[14] Mohd Mutalip S.S., Ab-Rahim S., Rajikin M.H. (2018). Vitamin E as an Antioxidant in Female Reproductive Health. Antioxidants.

[15] Fritsche S., Wang X., Jung C. (2017). Recent Advances in our Understanding of Tocopherol Biosynthesis in Plants: An Overview of Key Genes, Functions, and Breeding of Vitamin E Improved Crops. Antioxidants.

[16] Nogala-Kałucka M., Siger A. (2011). Tokochromanol-bioaktywne związki roślin oleistych. Od biosyntezy do biomarkerów. Rośliny Oleiste-Oilseed Crops.

[17] Zou L., Akoh C.C. (2015). Antioxidant activities of annatto and palm tocotrienol-rich fractions in fish oil and structured lipid-based infant formula emulsion. Food Chem..

[18] Khallouki F., Owen R.W., Akdad M., El Bouhali B., Silvente-Poirot S., Poirot M. (2020). Vitamin E: An overview. Molecular Nutrition.

[19] Krauß S., Darwisch V., Vetter W. (2018). Occurrence of tocopheryl fatty acid esters in vegetables and their non-digestibility by artificial digestion juices. Sci. Rep..

[20] Miyazawa T., Burdeos G.C., Itaya M., Nakagawa K., Miyazawa T. (2019). Vitamin E: Regulatory Redox Interactions. IUBMB Life.

[21] Niki E., Abe K., Niki E. (2019). Vitamin E: Structure, Properties and Functions. Vitamin E: Chemistry and Nutritional Benefits.

[22] Ranard K.M., Erdman J.W. (2018). Effects of dietary RRR α-tocopherol vs. all-racemic α-tocopherol on health outcomes. Nutr. Rev..

[23] Azzi A., Stocker A. (2000). Vitamin E: Non-antioxidant roles. Prog. Lipid Res..

[24] Wallert M., Börmel L., Lorkowski S. (2021). Inflammatory Diseases and Vitamin E—What Do We Know and Where Do We Go?. Mol. Nutr. Food Res..

[25] Institute of Medicine (2000). Dietary Reference Intakes for Vitamin C, Vitamin E, Selenium, and Carotenoids.

[26] Schmölz L., Birringer M., Lorkowski S., Wallert M. (2016). Complexity of vitamin E metabolism. World J. Biol. Chem..

[27] Yamanashi Y., Takada T., Kurauchi R., Tanaka Y., Komine T., Suzuki H. (2017). Transporters for the intestinal absorption of cholesterol, vitamin E, and vitamin K. J. Atheroscler. Thromb..

[28] Shen W.-J., Azhar S., Kraemer F.B. (2017). SR-B_1_: A Unique Multifunctional Receptor for Cholesterol Influx and Efflux. Annu. Rev. Physiol..

[29] Drotleff A.M., Bohnsack C., Schneider I., Hahn A., Ternes W. (2014). Human oral bioavailability and pharmacokinetics of tocotrienols from tocotrienol-rich (tocopherol-low) barley oil and palm oil formulations. J. Funct. Foods.

[30] Borel P., Desmarchelier C. (2016). Genetic Variations Involved in Vitamin E Status. Int. J. Mol. Sci..

[31] Schneider C. (2005). Chemistry and biology of vitamin E. Mol. Nutr. Food Res..

[32] Traber M.G., Kayden H.J. (1987). Tocopherol distribution and intracellular localization in human adipose tissue. Am. J. Clin. Nutr..

[33] Burton G.W., Traber M.G., Acuff R.V., Walters D.N., Kayden H., Hughes L., Ingold K.U. (1998). Human plasma and tissue α-tocopherol concentrations in response to supplementation with deuterated natural and synthetic vitamin E. Am. J. Clin. Nutr..

[34] Ikeda S., Toyoshima K., Yamashita K. (2001). Dietary sesame seeds elevate α- and γ-tocotrienol concentrations in skin and adipose tissue of rats fed the tocotrienol-rich fraction extracted from palm oil. J. Nutr..

[35] Jaafar F., Abdullah A., Makpol S. (2018). Cellular Uptake and Bioavailability of Tocotrienol-Rich Fraction in SIRT1-Inhibited Human Diploid Fibroblasts. Sci. Rep..

[36] Alcalá M., Sánchez-Vera I., Sevillano J., Herrero L., Serra D., Ramos M.P., Viana M. (2015). Vitamin E reduces adipose tissue fibrosis, inflammation, and oxidative stress and improves metabolic profile in obesity. Obesity.

[37] Yang C.S., Luo P., Zeng Z., Wang H., Malafa M., Suh N. (2020). Vitamin E and cancer prevention: Studies with different forms of tocopherols and tocotrienols. Mol. Carcinog..

[38] Traber M.G., Mah E., Leonard S.W., Bobe G., Bruno R.S. (2017). Metabolic syndrome increases dietary α-tocopherol requirements as assessed using urinary and plasma vitamin E catabolites: A double-blind, crossover clinical trial. Am. J. Clin. Nutr..

[39] Wong S.K., Kamisah Y., Mohamed N., Muhammad N., Masbah N., Fahami N.A.M., Mohamed I.N., Shuid A.N., Saad Q.M., Abdullah A. (2020). Potential role of tocotrienols on non-communicable diseases: A review of current evidence. Nutrients.

[40] Uchida T., Abe C., Nomura S., Ichikawa T., Ikeda S. (2012). Tissue distribution of α- And γ-tocotrienol and γ-tocopherol in rats and interference with their accumulation by α-tocopherol. Lipids.

[41] Patel V., Khanna S., Roy S., Ezziddin O., Sen C.K. (2006). Natural vitamin E α-tocotrienol: Retention in vital organs in response to long-term oral supplementation and withdrawal. Free Radic. Res..

[42] Uchida T., Nomura S., Oda H., Ikeda S. (2018). γ-Tocopherol Is Metabolized Faster than α-Tocopherol in Young Japanese Women. J. Nutr. Sci. Vitaminol..

[43] Traber M.G. (2013). Mechanisms for the prevention of vitamin e excess. J. Lipid Res..

[44] Ziegler M., Wallert M., Lorkowski S., Peter K. (2020). Cardiovascular and Metabolic Protection by Vitamin E: A Matter of Treatment Strategy?. Antioxidants.

[45] Azzi A. (2018). Many tocopherols, one vitamin E. Mol. Asp. Med..

[46] Kono N., Arai H. (2015). Intracellular Transport of Fat-Soluble Vitamins A and E. Traffic.

[47] Devaraj S., Leonard S., Traber M.G., Jialal I. (2008). Gamma-tocopherol supplementation alone and in combination with alpha-tocopherol alters biomarkers of oxidative stress and inflammation in subjects with metabolic syndrome. Free Radic. Biol. Med..

[48] Dutton P.J., Foster D.O., Burton G.W., Ingold K.U. (1990). Simon metabolites of α-tocopherol are not formed via a rate-controlling scission of the 3′C-H bond. Free Radic. Biol. Med..

[49] Birringer M., Drogan D., Brigelius-Flohe R. (2001). Tocopherols are metabolized in HepG2 cells by side chain ω-oxidation and consecutive β-oxidation. Free Radic. Biol. Med..

[50] Jiang Q. (2014). Natural forms of vitamin E: Metabolism, antioxidant, and anti-inflammatory activities and their role in disease prevention and therapy. Free Radic. Biol. Med..

[51] Birringer M., Lorkowski S. (2019). Vitamin E: Regulatory role of metabolites. IUBMB Life.

[52] Jiang Q. (2019). Natural forms of vitamin E and metabolites—Regulation of cancer cell death and underlying mechanisms. IUBMB Life.

[53] Schubert M., Kluge S., Schmölz L., Wallert M., Galli F., Birringer M., Lorkowski S. (2018). Long-chain metabolites of vitamin E: Metabolic activation as a general concept for lipid-soluble vitamins?. Antioxidants.

[54] Jiang Q., Yin X., Lill M.A., Danielson M.L., Freiser H., Huang J. (2008). Long- chain carboxychromanols, metabolites of vitamin E, are potent inhibitors of cyclooxygenases. Proc. Natl. Acad. Sci. USA.

[55] Jiang Q., Ames B.N. (2003). γ-Tocopherol, but not α-tocopherol, decreases proinflammatory eicosanoids and inflammation damage in rats. FASEB J..

[56] Newmark H.L., Huang M.-T., Reddy B.S. (2006). Mixed tocopherols inhibit azoxymethane-induced aberrant crypt foci in rats. Nutr. Cancer.

[57] Pein H., Ville A., Pace S., Temml V., Garscha U., Raasch M., Alsabil K., Viault G., Dinh C.-P., Guilet D. (2018). Endogenous metabolites of vitamin E limit inflammation by targeting 5-lipoxygenase. Nat. Commun..

[58] Rådmark O., Werz O., Steinhilber D., Samuelsson B. (2015). 5-Lipoxygenase, a key enzyme for leukotriene biosynthesis in health and disease. Biochim. Biophys. Acta Mol. Cell Biol. Lipids.

[59] Peters-Golden M., Henderson W.R. (2007). Leukotriens. N. Engl. J. Med..

[60] Wang D., DuBois R.N. (2010). Eicosanoids and cancer. Nat. Rev. Cancer.

[61] Martínez-Clemente M., Ferré N., González-Périz A., López-Parra M., Horrillo R., Titos E., Morán-Salvador E., Miquel R., Arroyo V., Funk C.D. (2010). 5-Lipoxygenase deficiency reduces hepatic inflammation and tumor necrosis factor α-induced hepatocyte damage in hyperlipidemia-prone apoe-null mice. Hepatology.

[62] Birringer M., Lington D., Vertuani S., Manfredini S., Scharlau D., Glei M., Ristow M. (2010). Proapoptotic effects of long-chain vitamin E metabolites in HepG2 cells are mediated by oxidative stress. Free Radic. Biol. Med..

[63] Jang Y., Park N.-Y., Rostgaard-Hansen A.L., Huang J., Jiang Q. (2016). Vitamin E metabolite 13′-carboxychromanols inhibit pro-inflammatory enzymes, induce apoptosis and autophagy in human cancer cells by modulating sphingolipids and suppress colon tumor development in mice. Free Radic. Biol. Med..

[64] Wallert M., Mosig S., Rennert K., Funke H., Ristow M., Pellegrino R.M., Cruciani G., Galli F., Lorkowski S., Birringer M. (2014). Long-chain metabolites of α-tocopherol occur in human serum and inhibit macrophage foam cell formation in vitro. Free Radic. Biol. Med..

[65] Ricciarelli R., Zingg J.-M., Azzi A. (2000). Vitamin E reduces the uptake of oxidized LDL by inhibiting CD36 scavenger receptor expression in cultured aortic smooth muscle cells. Circulation.

[66] Torquato P., Bartolini D., Giusepponi D., Saluti G., Russo A., Barola C., Birringer M., Galarini R., Galli F. (2016). a-13′-OH is the main product of a-tocopherol metabolism and influences CYP4F2 and PPAR? Gene expression in HepG2 human hepatocarcinoma cells. Free Radic. Biol. Med..

[67] Podszun M.C., Jakobi M., Birringer M., Weiss J., Frank J. (2017). The long chain α-tocopherol metabolite α-13′-COOH and γ-tocotrienol induce P-glycoprotein expression and activity by activation of the pregnane X receptor in the intestinal cell line LS 180. Mol. Nutr. Food Res..

[68] Barzegar-Amini M., Ghazizadeh H., Seyedi S.M.R., Sadeghnia H.R., Mohammadi A., Hassanzade-Daloee M., Barati E., Kharazmi-Khorassani S., Kharazmi-Khorassani J., Mohammadi-Bajgiran M. (2019). Serum vitamin E as a significant prognostic factor in patients with dyslipidemia disorders. Diabetes Metab. Syndr. Clin. Res. Rev..

[69] Godoy-Parejo C., Deng C., Zhang Y., Liu W., Chen G. (2020). Roles of vitamins in stem cells. Cell. Mol. Life Sci..

[70] Maniam S., Mohamed N., Shuid A.N., Soelaiman I.N. (2008). Palm tocotrienol exerted better antioxidant activities in bone than α-tocopherol. Basic Clin. Pharmacol. Toxicol..

[71] Wong R.S.Y., Radhakrishnan A.K. (2012). Tocotrienol research: Past into present. Nutr. Rev..

[72] Packer L., Weber S.U., Rimbach G. (2001). Molecular aspects of α-tocotrienol antioxidant action and cell signalling. J. Nutr..

[73] Zingg J.-M., Azzi A. (2004). Non-Antioxidant Activities of Vitamin E. Curr. Med. Chem..

[74] Traber M.G., Atkinson J. (2007). Vitamin E, antioxidant and nothing more. Free Radic. Biol. Med..

[75] Wu D., Meydani S.N., Weber P., Birringer M., Blumberg J.B., Eggersdorfer M., Frank J. (2019). Vitamin E, Immune Function, and Protection Against Infection. Vitamin E in Human Health.

[76] Iddir M., Brito A., Dingeo G., Fernandez Del Campo S.S., Samouda H., La Frano M.R., Bohn T. (2020). Strengthening the immune system and reducing inflammation and oxidative stress through diet and nutrition: Considerations during the covid-19 crisis. Nutrients.

[77] Lee G.Y., Han S.N. (2018). The role of vitamin E in immunity. Nutrients.

[78] Traber M.G., Sokol R.J., Burton G.W., Ingold K.U., Papas A.M., Huffaker J.E., Kayden H.J. (1990). Impaired ability of patients with familial isolated vitamin E deficiency to incorporate α-tocopherol into lipoproteins secreted by the liver. J. Clin. Investig..

[79] Azzi A. (2007). Molecular mechanism of α-tocopherol action. Free Radic. Biol. Med..

[80] Shibata A., Kobayashi T., Asai A., Eitsuka T., Oikawa S., Miyazawa T., Nakagawa K. (2017). High purity tocotrienols attenuate atherosclerotic lesion formation in apoE-KO mice. J. Nutr. Biochem..

[81] Abraham A., Kattoor A.J., Saldeen T., Mehta J.L. (2019). Vitamin E and its anticancer effects. Crit. Rev. Food Sci. Nutr..

[82] Constantinou C., Charalambous C., Kanakis D. (2020). Vitamin E and cancer: An update on the emerging role of γ and δ tocotrienols. Eur. J. Nutr..

[83] Wu G., Zhu H., Wu X., Liu L., Ma X., Yuan Y., Fu X., Zhang L., Lv Y., Li D. (2020). Anti-allergic function of α-Tocopherol is mediated by suppression of PI3K-PKB activity in mast cells in mouse model of allergic rhinitis. Allergol. Immunopathol..

[84] Ramanathan N., Tan E., Loh L.J., Soh B.S., Yap W.N. (2018). Tocotrienol is a cardioprotective agent against ageing-associated cardiovascular disease and its associated morbidities. Nutr. Metab..

[85] Shibata A., Kawakami Y., Kimura T., Miyazawa T., Nakagawa K. (2016). α-Tocopherol Attenuates the Triglyceride- and Cholesterol-Lowering Effects of Rice Bran Tocotrienol in Rats Fed a Western Diet. J. Agric. Food Chem..

[86] Tan S.M.Q., Chiew Y., Ahmad B., Kadir K.A. (2018). Tocotrienol-rich vitamin E from palm oil (Tocovid) and its effects in diabetes and diabetic nephropathy: A pilot phase II clinical trial. Nutrients.

[87] Baradaran A., Nasri H., Rafieian-Kopaei M. (2014). Oxidative stress and hypertension: Possibility of hypertension therapy with antioxidants. J. Res. Med. Sci..

[88] Wong W.-Y., Ward L.C., Fong C.W., Yap W.N., Brown L. (2017). Anti-inflammatory γ- and δ-tocotrienols improve cardiovascular, liver and metabolic function in diet-induced obese rats. Eur. J. Nutr..

[89] Azzi A. (2017). Antioxidants: Wonder drugs or quackery?. Biofactors.

[90] Khadangi F., Azzi A. (2019). Vitamin E—The Next 100 Years. IUBMB Life.

[91] Azzi A. (2019). Tocopherols, tocotrienols and tocomonoenols: Many similar molecules but only one vitamin E. Redox Biol..

[92] Nordic Nutrition Recommendations (2014). Integrating Nutrition and Physical Activity.

[93] Jarosz M., Rychlik E., Stoś K., Charzewska J. (2020). Dietary References for Polish Population and Their Application.

[94] Deutschland-Austria-Confoederatio Helvetica (2015). Deutsche Gesellschaft fur Ernahrung, Osterreichische Gesellschaft fur Ernahrung, Schweizerische Gesellschaft fur Ernahrung, Referenzwerte fur die Nahrstoffzufuhr.

[95] Eggersdorfer M. (2017). Global vitamin status—Perspectives for nutrition. Free Radic. Biol. Med..

[96] Raederstorff D., Wyss A., Calder P.C., Weber P., Eggersdorfer M. (2015). Vitamin E function and requirements in relation to PUFA. Br. J. Nutr..

[97] Dror D.K., Allen L.H. (2011). Vitamin e deficiency in developing countries. Food Nutr. Bull..

[98] Traber M.G. (2014). Vitamin E inadequacy in humans: Causes and consequences. Adv. Nutr..

[99] Huang J., Weinstein S.J., Yu K., Männistö S., Albanes D. (2019). Relationship Between Serum Alpha-Tocopherol and Overall and Cause-Specific Mortality. Circ. Res..

[100] Torquato P., Marinelli R., Bartolini D., Galli F. (2020). Vitamin E: Nutritional aspects. Molecular Nutrition.

[101] Wright M.E., Lawson K.A., Weinstein S.J., Pietinen P., Taylor P.R., Virtamo J., Albanes D. (2006). Higher baseline serum concentrations of vitamin E are associated with lower total and cause-specific mortality in the Alpha-Tocopherol, Beta-Carotene Cancer Prevention Study. Am. J. Clin. Nutr..

[102] Mangialasche F., Xu W., Kivipelto M., Costanzi E., Ercolani S., Pigliautile M., Cecchetti R., Baglioni M., Simmons A., Soininen H. (2012). Tocopherols and tocotrienols plasma levels are associated with cognitive impairment. Neurobiol. Aging.

[103] Hamułka J., Górnicka M., Sulich A., Frąckiewicz J. (2019). Weight loss program is associated with decrease α-tocopherol status in obese adults. Clin. Nutr..

[104] Kabat G.C., Heo M., Ochs-Balcom H.M., Leboff M.S., Mossavar-Rahmani Y., Adams-Campbell L.L., Nassir R., Ard J., Zaslavsky O., Rohan T.E. (2016). Longitudinal association of measures of adiposity with serum antioxidant concentrations in postmenopausal women. Eur. J. Clin. Nutr..

[105] Wallström P., Wirfält E., Lahmann P.H., Gullberg B., Janzon L., Berglund G. (2001). Serum concentrations of β-carotene and α-tocopherol are associated with diet, smoking, and general and central adiposity. Am. J. Clin. Nutr..

[106] Öhrvall M., Tengblad S., Vessby B. (1993). Lower tocopherol serum levels in subjects with abdominal adiposity. J. Intern. Med..

[107] Stenzel A.P., Carvalho R., Jesus P., Bull A., Pereira S., Saboya C., Ramalho A. (2018). Serum antioxidant associations with metabolic characteristics in metabolically healthy and unhealthy adolescents with severe obesity: An observational study. Nutrients.

[108] Shahinfar H., Akbarzade Z., Djafari F., Shab-Bidar S. (2020). Association of nutrient patterns and metabolic syndrome and its components in adults living in Tehran, Iran. J. Diabetes Metab. Disord..

[109] Chai W., Conroy S.M., Maskarinec G., Franke A.A., Pagano I.S., Cooney R.V. (2010). Associations between obesity and serum lipid-soluble micronutrients among premenopausal women. Nutr. Res..

[110] Goncalves A., Amiot M.-J. (2017). Fat-soluble micronutrients and metabolic syndrome. Curr. Opin. Clin. Nutr. Metab. Care.

[111] Geiker N.R.W., Veller M., Kjoelbaek L., Jakobsen J., Ritz C., Raben A., Astrup A., Lorenzen J.K., Larsen L.H., Bügel S. (2018). Effect of low energy diet for eight weeks to adults with overweight or obesity on folate, retinol, vitamin B12, D and e status and the degree of inflammation: A post hoc analysis of a randomized intervention trial. Nutr. Metab..

[112] Pang K.-L., Chin K.-Y. (2019). The role of tocotrienol in protecting against metabolic diseases. Molecules.

[113] Uto-Kondo H., Ohmori R., Kiyose C., Kishimoto Y., Saito H., Igarashi O., Kondo K. (2009). Tocotrienol suppresses adipocyte differentiation and Akt phosphorylation in 3T3-L1 preadipocytes. J. Nutr..

[114] Ima-Nirwana S., Suhaniza S. (2004). Effects of Tocopherols and Tocotrienols on Body Composition and Bone Calcium Content in Adrenalectomized Rats Replaced with Dexamethasone. J. Med. Food.

[115] Zhao L., Fang X., Marshall M.R., Chung S. (2016). Regulation of obesity and metabolic complications by gamma and delta tocotrienols. Molecules.

[116] Agarwal S., Reider C., Brooks J.R., Fulgoni V.L. (2015). Comparison of Prevalence of Inadequate Nutrient Intake Based on Body Weight Status of Adults in the United States: An Analysis of NHANES 2001–2008. J. Am. Coll. Nutr..

